# Integrating mental health literacy into Chinese college student mental health education in the post-COVID-19 era

**DOI:** 10.3389/fpubh.2024.1456579

**Published:** 2024-09-26

**Authors:** Tao Gao, Bo Gao, Linzhao Wang, Zaihua Qing

**Affiliations:** ^1^Guangdong Industry Polytechnic University, Guangzhou, China; ^2^Guangdong Ocean University, Zhanjiang, Guangdong, China; ^3^Hunan University of Finance and Economics, Changsha, Hunan, China

**Keywords:** mental health literacy, mental health education, China, college student, COVID-19

## Abstract

This study investigates the influence of COVID-19 on the mental health of Chinese college students and evaluates the current status of mental health literacy (MHL) education. With mental health issues among students becoming increasingly prominent due to the pandemic and rapid social changes, enhancing MHL is critical. The research highlights the necessity of integrating mental health education with MHL to foster resilience and effective coping strategies. It proposes a model combining online and offline education to maximize accessibility and engagement. By examining both domestic and international perspectives, the study underscores the importance of comprehensive mental health education reform in the post-pandemic era. This integrated approach aims to improve students’ mental well-being, reduce stigma, and encourage help-seeking behaviors, ultimately contributing to a healthier and more supportive campus environment.

## Introduction

In recent years, enhancing the mental health literacy (MHL) of the Chinese population has become a key strategy for national development (1). In 2021, the General Office of the Ministry of Education issued a directive to strengthen the management of student mental health. This notice emphasized the importance of “improving the pertinence and effectiveness of student mental health work, strengthening professional support and scientific management, and focusing on improving student MHL,” highlighting the necessity of “strengthening source management and comprehensively improving student MHL” (2). In 2023, the Ministry of Education, along with 17 other departments, released the Special Action Plan for Comprehensively Strengthening and Improving the Mental Health of Students in the New Era (2023–2025). This document underscored that “with rapid economic and social development, students’ growth environments are continuously changing, and the impact of the COVID-19 pandemic has exacerbated students’ mental health problems.” It clearly called for “comprehensive efforts to strengthen and improve the mental health of students in the new era and enhance their MHL” (3).

COVID-19 has been a significant global public health emergency (4). While the majority of infected individuals experience mild symptoms such as fatigue, coughing, and headaches, up to 20% can develop severe complications like pneumonia, respiratory failure, and even death (5, 6). In response, countries worldwide have implemented unprecedented measures to control the virus’s spread. These include canceling large gatherings, closing schools, limiting public interactions, and promoting social isolation and remote work (7). Additionally, efforts such as early screening, contact tracing, monitoring, and both preventive and mandatory isolation have been enforced for suspected and confirmed cases (8).

The pandemic has also triggered widespread emotional responses, including fear, anger, stigmatization, low self-esteem, and loss of self-control, which have further exacerbated mental health challenges (9–11). Consequently, there has been an increase in serious mental health issues such as depression, anxiety, psychological distress, fatigue, panic attacks, post-traumatic stress disorder (PTSD), and insomnia (12–16). College students, in particular, have shown heightened levels of negative emotions like anxiety, panic, and interpersonal sensitivity, contributing to a rise in psychological problems such as depression, anxiety, and PTSD (17, 18). In some cases, these challenges have even led to extreme outcomes like suicide, though these instances remain relatively rare (19).

Despite the severity of these mental health issues, many individuals have not sought treatment (20, 21). In China, for example, 63% of suicide cases are linked to mental health problems, yet only 7% of these individuals received professional mental health support before their death (22). Various factors contribute to this, including societal neglect, apathy, and discrimination against individuals with mental illnesses, which significantly hinder their access to care, rehabilitation, education, employment, and social participation (23). This situation underscores the critical need to improve public mental health literacy (MHL) (24).

MHL was initially defined as the ability to “help people recognize, understand, and prevent knowledge and concepts related to mental illness” (25). Some researchers later expanded the concept of MHL into three key dimensions: recognition, knowledge, and attitude (26). As research has advanced, scholars have increasingly incorporated elements that promote mental health within the MHL framework (27). Building on earlier studies, Jiang and his colleagues defined MHL as “an individual’s knowledge, attitudes, and behaviors in promoting their own and others’ mental health and coping with mental illness” (28). Research on MHL, both domestically and internationally, has primarily focused on recognizing mental illnesses, enhancing mental health knowledge, addressing stigmatizing attitudes, and encouraging help-seeking behaviors (25–27).

Given that Chinese university students represent a densely populated group, it is crucial to prevent psychological problems triggered by sudden public health emergencies. Improving college students’ mental health is significantly influenced by MHL. As an emerging research field, timely reform of university MHL education is essential in the post-pandemic era. This is vital for enhancing college students’ mental health and mitigating the impact of sudden public health incidents on their mental well-being.

## The influence of COVID-19 on Chinese mental health and the research status of psychological literacy education

Research on the psychological impact of COVID-19 is still in its early stages. For college students, a highly concentrated group, understanding their psychological state under the impact of the epidemic is crucial. Ye’s research (29) found that 13.2% of college students exhibited emotions such as nervousness, fear, sadness, and worry in response to COVID-19. Under the normalization of epidemic prevention and control, the developmental psychological crises faced by young college students have become more complex and numerous than in previous years. Mental health surveys have shown a significant year-on-year increase in emotions such as anxiety, panic, depression, loneliness, and boredom. The severe domestic epidemic prevention and control situation at that time, along with the worsening global pandemic, brought enormous psychological pressure to young students studying on campus.

Addressing psychological problems caused by the epidemic can be alleviated by improving students’ MHL. The concept of integrated MHL has only emerged in recent years, resulting in limited research conducted within this framework (24). Regarding college students’ psychological literacy during COVID-19, the relevant literature is still sparse. As of June 28, 2024, only 11 articles on the topic of “Mental Health Literacy and the Epidemic” were found on the prominent Chinese journal website China Knowledge Network, and only 7 of these articles focused on college students as research subjects.

Domestic scholars began researching the identification of mental illnesses relatively late, with a focus on the awareness rate of mental health knowledge. In 2015, the awareness rate of mental health knowledge among urban residents in China was just 28.12%, far below the 80% target set by the “National Guidelines for the Development of the Mental Health Work System (2008–2015)” (30). A study conducted by Li and his colleagues (31) examined the MHL of urban residents and the factors influencing it, revealing that respondents generally held strong negative attitudes toward individuals with mental disorders.

Several studies indicate that the Chinese public often perceives those with mental illnesses as having unpredictable behavior, needing special care, and possessing personality flaws, with many exhibiting behavior seen as unsettling, dangerous, or embarrassing (32, 33). Rural residents in China, on the other hand, show even lower awareness of common mental illnesses and demonstrate more pronounced discriminatory attitudes toward those affected (34, 35). During the COVID-19 pandemic, college students with higher levels of MHL were more inclined to seek or recommend professional assistance (36). Generally, the public’s literacy regarding the promotion of mental health is higher than their literacy in managing mental illnesses, and their capacity for self-care surpasses that of helping others (24).

In summary, although China has made some progress in MHL education, the overall level still needs improvement. Especially in the post-COVID-19 era, it has become crucial to comprehensively strengthen MHL education, help college students cope with psychological crises, and improve their mental health.

## The impact of COVID-19 on people’s mental health in other countries and the research status of psychological literacy education

The World Health Organization declared COVID-19 a global pandemic, prompting many higher education institutions to implement measures to ensure student safety. Psychologically and socially, COVID-19 has had a particularly severe impact on young people in 46 countries, leading to an increase in severe depression and generalized anxiety disorder, significantly affecting students’ mental health (37). According to Morneau Shepell’s Mental Health Index, the pandemic led to a 16% decrease in mental health levels in Canada (38).

Research in Mexico has shown that students experience significant stress due to changes in curriculum patterns, leading to mental health issues such as stress, anxiety, insomnia, and worry (39, 40). A study in the United States indicates differences between undergraduate and graduate students regarding mental health and happiness related to COVID-19. Compared to graduate students, undergraduate students reported higher levels of stress perception, more repetitive negative thoughts, less positive attitudes, and less teacher support (41).

Research on psychological literacy abroad has found that the recognition rate of different types of psychological disorders varies among the public in different countries and generally shows a trend of increasing with economic development (24). For instance, in Portugal, the depression recognition rate among young people is 27.2% (42). In Germany, public awareness of depression and schizophrenia has risen over an eight-year period to 37.5 and 22.4%, respectively (43). In the United States, the public’s ability to recognize depression is notably higher, with a rate of 58.5% (44).

The study also revealed that stigmatizing attitudes toward mental illness remain relatively stable across different cultures and time periods (22). In Europe and the United States, there is a widespread perception that individuals with mental illnesses are dangerous, unpredictable, and unlikely to recover, which leads to social exclusion (45, 46). Although MHL has improved among the German public, there has been little progress in the social acceptance of those with mental illnesses (43).

Globally, attitudes toward mental health support tend to be negative (47). In many Asian cultures, mental illness is not only seen as a burden on individuals and society but also as a source of family shame (48). According to World Health Organization data, 50.3% of severe mental illness patients in developed countries and 85.4% in developing countries did not receive treatment in the year before the survey (49). Furthermore, 30% of the European public believe that professional psychological help might not only be ineffective but could even exacerbate the situation (50).

Despite these challenges, public understanding of mental illness and the effectiveness of professional treatment has gradually improved over the past few decades. For example, in Australia, public confidence in the effectiveness of psychiatrists and psychologists has significantly increased over the past 10 years (51). However, this increased awareness has not led to a corresponding rise in the number of people seeking professional treatment (21).

Overall, international research on MHL underscores the considerable potential for enhancing public MHL. This is crucial for improving mental health outcomes and supporting others (24, 52). While individual differences in knowledge and concepts within MHL are notable, variations in attitudes and behavioral habits are less pronounced. Interventions can more readily alter knowledge and concepts, but changing deeply ingrained attitudes and behaviors remains a greater challenge (1, 53, 54). Therefore, current efforts should prioritize strengthening MHL, particularly in how individuals manage and respond to mental illness in others. In-depth research on the conditions and mechanisms for changing attitudes and habits related to mental health is needed to enhance college students’ psychological literacy.

## Constructing a model for integrating mental health education with mental health literacy education for college students

COVID-19 has greatly impacted various sectors, including education and interpersonal relationships, posing significant challenges for Chinese college students. Mental health intervention faces new challenges as well. Psychological literacy has become an important indicator affecting mental health status and development. One of the key tasks is to examine mental health education from the new perspective of psychological literacy.

## Background and necessity

With the rising prevalence of mental health issues among adolescents, it is essential to develop innovative methods to support their mental well-being (52). Psychological health education and support measures within school environments can have long-term positive effects on psychological, social, and behavioral development (55–58). MHL education involves identifying mental health issues, disseminating mental health knowledge, implementing resilience-building strategies, and encouraging appropriate help-seeking behavior (59). MHL encompasses the ability to identify mental health issues, knowledge of mental health, resilience strategies, and appropriate seeking behavior (26, 53, 60, 61). Although research in Australia is limited (62), studies in the United States, Canada, and Norway have demonstrated that school-based projects positively impact MHL (62–64).

## Current situation and challenges

The mental health of college students is crucial for their personal development, academic achievement, and social adaptation (55–58). However, the mental health problems of college students are becoming increasingly prominent due to the acceleration of social development and increased life pressure, making it a significant social concern. Young people need comprehensive MHL to support their own and others’ mental health. Future research must focus on incorporating MHL into current mental health education curricula to address the needs of young people (52).

Chinese scholars have made progress in integrating psychological literacy education into mental health education (65). Although still in the early stages of exploration, feasible methods for improving MHL have been identified. Most Chinese universities have made mental health education a compulsory course, but further efforts are needed to establish a separate course on MHL. The best way to enhance students’ psychological literacy is to integrate psychological health literacy knowledge with mental health education comprehensively, thereby improving their psychological quality and coping abilities.

## Integrating psychological health education and literacy knowledge for college students

Research on the integration of mental health education and psychological literacy among college students is still in its infancy (66). Psychological literacy is foundational; if the root is strong, the tree of mental health can flourish. The two are inseparable and mutually influential, requiring a connected and developmental perspective to promote research. In the future, there will inevitably be pandemics of unknown viruses again. Based on the impact of COVID-19 on college students’ mental health and mental literacy, it is important to consider the implications of the COVID-19 epidemic for China’s MHL education reform strategy. This will provide important insights for mental health education reform in China and globally.

Students with high MHL often have taken at least one course related to clinical psychology (67). Research shows that participation in psychology courses helps form and improve the MHL of college students (68). Integrating MHL knowledge with mental health education for college students can comprehensively enhance their psychological quality and coping abilities.

## Comprehensive knowledge system construction

Integrating concepts of healthy living, mental health maintenance, and the identification and response to mental illness into the teaching content of mental health courses can help students establish a scientific understanding of mental illness. Addressing the lack of knowledge in identifying and coping with psychological disorders, the focus should be on the following content:

Recognizing typical symptoms of common mental disorders, including schizophrenia, depression, anxiety, and bipolar disorder, is crucial. It’s important to understand methods for timely detection of one’s own psychological state and to be familiar with self-adjustment and help-seeking strategies for managing psychological disorders. Additionally, being aware of others’ psychological states and knowing how to accept and assist them is essential for fostering a supportive environment.

By improving students’ recognition of their own and others’ mental illnesses, enhancing their daily mental health care awareness, reducing prejudice against mental illnesses, and promoting self-help and helping others, the course can have a comprehensive impact.

## Combining prevention and intervention

The educational content should integrate both prevention and intervention strategies. It’s essential to impart knowledge on mental health prevention, including stress management, emotional regulation, and self-awareness. Simultaneously, students should learn intervention methods for psychological issues, such as coping strategies for anxiety and depression, psychological counseling, and treatment approaches. This comprehensive approach enables students to prevent psychological problems before they occur and to intervene promptly when issues arise.

## Reform of the integration of psychological health education for college students and the education form of psychological health literacy

### Personalized teaching methods

Develop flexible and diverse teaching approaches tailored to the different needs and characteristics of students. For example, combine case analysis, group discussions, and role-playing to engage students actively, stimulate their interest and motivation, and improve learning effectiveness.

### Practical learning activities

Conduct practical activities such as MHL knowledge competitions, themed lectures, and psychological counseling services. These activities enable students to apply theoretical knowledge to real life and cultivate problem-solving and practical skills.

### Increasing contact experience

Both direct and indirect contact with individuals who have mental illnesses has been shown to significantly reduce stigma (14). To enhance students’ contact experience and foster greater understanding of mental health issues, the following methods should be employed:

First, utilizing direct contact methods involves inviting individuals with mental illnesses to share their personal experiences and coping strategies during mental health classes or psychological lectures. This firsthand interaction allows students to gain valuable insights into the challenges faced by those with mental health conditions. Additionally, organizing volunteer activities related to psychological care provides students with opportunities for real-life interactions with individuals undergoing treatment. These experiences help to humanize mental health issues, challenge stereotypes, and ultimately reduce stigma within the student community.

Second, employing indirect contact methods includes using personal narrative videos, documentaries, and films featuring individuals who have experienced mental illnesses. These media formats effectively convey mental health knowledge by showcasing positive interactions between individuals with mental illnesses and the general public. By portraying authentic stories and perspectives, these materials promote empathy, understanding, and acceptance among students who may not have direct personal contact with individuals facing mental health challenges.

Incorporating both direct and indirect contact methods into mental health education not only enriches students’ learning experiences but also contributes to a more supportive and inclusive campus environment. These approaches not only educate students about mental health but also empower them to actively combat stigma through increased awareness and compassionate engagement.

## Promoting mental health service resources

Encourage students to seek professional psychological help by promoting on-campus and off-campus mental health service resources. Studies have found that those who have not sought help before often feel more shame and prefer to solve problems on their own (16). Focus on informing these students about available resources to reduce doubts and shame about professional assistance. During public health crises like COVID-19, when loneliness and mental health issues are prevalent (69), it is crucial to strengthen the promotion of mental health services to increase students’ willingness to seek help.

## Evaluation and adjustment

Establish a comprehensive evaluation mechanism to regularly assess the educational models, collect feedback from students and teachers, and adjust the content and methods as needed. Continuous evaluation and adjustment ensure the effectiveness and improvement of the education model.

## Implementing a hybrid approach to mental health education and literacy

During the COVID-19 pandemic, more and more people worldwide faced restrictions and adhered to social isolation recommendations (70). These measures brought mental health risks, as unpredictable viruses and destructive events due to climate change became increasingly common (71). During this period, online teaching rapidly gained popularity and developed to minimize disruptions to students’ learning (72). Looking ahead, the mental health care sector is expected to shift toward online prevention, treatment, and care (73). Currently, timely exploration of integrating online and offline mental health and literacy education can better prepare for emergencies and has significant practical implications.

In the post-COVID-19 era, the combination of online and offline education will become a trend, offering a new opportunity for integrating mental health education and MHL. Utilizing both methods comprehensively can better meet the diverse learning needs of college students, improving the coverage and effectiveness of education.

## The advantages of online education

Online education can transcend time and space limitations, providing students with flexible learning options. Through online courses, virtual classrooms, and digital resources, students can access mental health knowledge and literacy education content anytime and anywhere. Examples include online mental health lectures, micro-courses on MHL, and psychological knowledge competitions, which not only enrich learning formats but also boost student participation and enthusiasm.

## The advantages of offline education

Offline education emphasizes interactivity and practicality. Face-to-face communication and hands-on activities help students gain a deeper understanding and mastery of mental health knowledge. Offline mental health courses, group counseling, psychological workshops, and role-playing activities offer real learning situations, promote emotional communication and experience sharing among students, and enhance learning effectiveness.

## Combining online and offline education models

Integrating online and offline education maximizes their respective advantages and forms a complementary approach. Specific measures include:

1. Blended Curriculum Design: combining online self-directed learning with offline classroom teaching. For instance, students can preview course content through online platforms and engage in in-depth discussions and case studies in the classroom, merging theory with practice.
2. Flexible Learning Arrangements: flexibly arranging online and offline learning time based on students’ actual situations. Online teaching can be used for highly theoretical knowledge, while offline teaching can be arranged for content requiring interaction and practice.
3. Interaction and Feedback Mechanism: collecting student learning feedback through online platforms and adjusting teaching content and methods in a timely manner. Meanwhile, offline teaching allows teachers to communicate face-to-face, understanding students’ psychological states and learning needs, and providing targeted guidance and support.

In summary, integrating online and offline mental health education and MHL education can fully leverage modern educational technology and resources, offering flexible, diverse, and effective educational methods. This comprehensive model can enhance college students’ MHL, promote their overall development and social adaptability, and lay a solid foundation for building a healthy and harmonious campus environment.

## Conclusion

Enhancing the MHL of college students through an integrated model of mental health education and MHL is crucial for improving their overall mental well-being. This integrated approach aims to holistically improve students’ psychological resilience and coping skills by seamlessly combining mental health education with MHL. This comprehensive, systematic, and effective platform offers both online and offline teaching methods to ensure accessibility and engagement.

The COVID-19 pandemic has underscored the importance of MHL, revealing significant gaps in mental health awareness, stigma reduction, and help-seeking behaviors. By addressing these gaps, this model helps students better navigate social life, supports their personal development, and prepares them to handle future public health emergencies more effectively.

Integrating mental health education and MHL is not only vital for enhancing students’ MHL but also serves as a valuable reference for future educational reforms. This holistic education model plays a significant role in promoting MHL, equipping students with the necessary tools for both academic and personal success, and setting a precedent for innovative educational strategies that can enhance the well-being of students in various educational contexts.

## Data Availability

The raw data supporting the conclusions of this article will be made available by the authors, without undue reservation.
